# Serum Metabolic Profile in Multiple Sclerosis Patients

**DOI:** 10.1155/2011/167156

**Published:** 2011-06-28

**Authors:** Barbara Tavazzi, Anna Paola Batocchi, Angela Maria Amorini, Viviana Nociti, Serafina D'Urso, Salvatore Longo, Stefano Gullotta, Marika Picardi, Giuseppe Lazzarino

**Affiliations:** ^1^Institute of Biochemistry and Clinical Biochemistry, Catholic University of Rome, 00168 Rome, Italy; ^2^Institute of Neurology, Catholic University of Rome, 00168 Rome, Italy; ^3^Department of Biology, Geology and Environmental Sciences, Division of Biochemistry and Molecular Biology, University of Catania, 95125 Catania, Italy

## Abstract

Multiple sclerosis (MS) is a progressive demyelinating process considered as an autoimmune disease, although the causes of this pathology have not been yet fully established. Similarly to other neurodegenerations, MS is characterized by a series of biochemical changes affecting to different extent neuronal functions; great attention has been given to oxidative/nitrosative stress and to alterations in mitochondrial functions. According to previous data, MS patients show significant changes in the circulating concentrations of different metabolites, although it is still unclear whether uric acid undergoes to decrease, increase, or no change under this pathological condition. In this study, we report the serum metabolic profile in terms of purines, pyrimidines, creatinine, malondialdehyde, ascorbic acid, nitrite, and nitrate in a group of 170 MS patients. The results show increase in circulating uric acid and other oxypurines (hypoxanthine and xanthine), as well as in uridine and *β*-pseudouridine. The concomitant increase in circulating creatinine, malondialdehyde, nitrite, and nitrate, and decrease in ascorbic acid, demonstrates that MS induces alteration in energy metabolism and in oxidants/antioxidants balance that can be monitored in serum of MS patients.

## 1. Introduction

Multiple sclerosis (MS) is a progressive, invalidating pathological state, the exact etiology of which is still uncertain [1]. It is considered as an autoimmune disease although the reasons for the autoimmune demyelinization are far to be clear [2]. At the molecular level, MS is characterized by a series of biochemical changes affecting neuronal functions [3], some of which are in common with other neurodegenerations such as Alzheimer's [4] and Parkinson's diseases [5]. Particularly, one of these common features is the neuronal imbalance in oxidants/antioxidants, with reactive oxygen species (ROS) and reactive nitrogen species (RNS) as the excess oxidants [6, 7] and uric acid as the putative defective antioxidant [8–11]. Recently, mitochondrial malfunctioning has been indicated to play a central role in the overall derangement of brain metabolism observed in MS [12]. The consequences of mitochondrial perturbation are critical for the correct functioning of the electron transport chain coupled to oxidative phosphorylation and, hence, for the maintenance of the cell energy homeostasis. Furthermore, the abundant literature has linked mitochondrial dysfunction with ROS overflow [13]. If the imbalance in energy production and consumption is operative, that is, the amount of ATP produced does not satisfy the cell energy demand, it is unavoidable that the purine nucleotide degradation pathway is activated. This provokes an increased generation of nucleosides (adenosine, guanosine, and inosine) and oxypurines (hypoxanthine, xanthine, and uric acid), which can freely cross the cell membrane being released in part in the extracellular space. In the brain tissue, this phenomenon contributes to the significant increase of these compounds in the cerebrospinal fluid (CSF) observed under different pathological states [14, 15], including MS [16].

Permeable metabolites generated in excess by transient or chronic dysfunction of brain metabolism are sooner or later found into the blood stream, potentially contributing to a significant raise over their respective circulating physiological levels [17, 18]. Therefore, several low-molecular-weight compounds can be good candidate as potential blood biomarkers of neurodegeneration. According to the present knowledge, it is conceivable that compounds deriving from ROS and RNS overproduction and metabolites generated by altered energy metabolism might be detected in excess in blood samples from MS patients, possibly being valid predictors of the disease evolution. This logical cause-effect link has been proven for several compounds related to ROS and RNS overproduction, so that increase in circulating nitric oxide (NO) products [19, 20] and increase in lipid peroxidation products [21] have been found in plasma/serum of MS patients. Surprisingly, this does not seem to apply to products deriving from the imbalance of energy metabolism since several studies indicated significant decrease in plasma/serum concentrations of uric acid (the end product of purine nucleotide catabolism) in MS patients [22–25]. The rather improbable explanation for this fact is that brain uric acid, acting as a potent NO scavenger, is oxidized in consequence of the increased NO generation. The final result would be a significant decrease in uric acid circulating levels. In contrast to these results, a number of clinical studies have indicated either no change [26, 27] or increase [28, 29] in plasma/serum uric acid of MS patients, thereby rendering unclear whether this compound is modified under this pathological condition. Recently, we reported a concomitant increase in the plasma and CSF concentrations not only of uric acid but also of other oxypurines and nucleosides in a cohort of MS patients [16, 30].

To reinforce our previous results, we here report the metabolic profile in terms of purines, pyrimidines, creatinine, malondialdehyde (MDA), ascorbic acid, nitrite, and nitrate determined in a group of 170 MS patients. Concentrations of the various metabolites were compared with those recorded in a group of 163 healthy controls. In order to have indications on the potential clinical utility of the routine metabolic profiling of MS patients, metabolite changes were analyzed for a correlation with the severity of the disease and MS subtypes.

## 2. Materials and Methods

### 2.1. Selection and Clinical Evaluation of the Patients

One hundred and seventy MS patients were included in this study. They were assessed clinically at the Institute of Neurology of the “Policlinico Gemelli” of the Catholic University of Rome, using the Extended Disability Status Scale score (EDSS) [31]. Patients were classified into relapsing remitting (RR), secondary progressive (SP), or primary progressive (PP), according to what described elsewhere [32]. The control group consisted of 163 healthy subjects, matched for age and gender, and recruited among the personnel of the two Universities undergoing the annual health checkup. All selected subjects had no acute or chronic pathologies. The study was approved by the local Ethic Committee. Written informed consents were obtained.

### 2.2. Preparation of Samples for the Serum Metabolic Profiling

In both patients and controls, peripheral venous blood samples were collected from the antecubital vein into VACUETTE polypropylene tubes containing serum separator and clot activator (Greiner-Bio One GmbH, Kremsmunster, Austria). After 40 minutes at room temperature, samples were centrifuged at 1890 ×g for 10 min to separate sera. Aliquots were first diluted with doubly-distilled water (1 : 2, v : v) and then deproteinized by ultrafiltration, according to a procedure described in detail elsewhere [33]. The deproteinized ultrafiltrate fluid was used to quantify the metabolites of interest using a single, ion-pairing, high-performance liquid chromatographic (HPLC) analysis which allows the simultaneous isocratic separation of creatinine, purines (hypoxantine, xanthine, uric acid, inosine, guanosine), pyrimidines (uracil, *β*-pseudouridine, thymine, uridine, thymidine, orotic acid), ascorbic acid, MDA, nitrite, and nitrate [33].

Deproteinized samples were loaded (200 *μ*L) onto a Hypersil C-18, 250 × 4.6 mm, 5 *μ*m particle size column, provided with its own guard column (ThermoFisher Italia, Rodano, Milan, Italy). The chromatographic column was connected to an HPLC apparatus consisting of a SpectraSystem P4000 pump system and a highly-sensitive UV6000LP diode array detector (ThermoFisher Italia, Rodano, Milan, Italy), equipped with a 5 cm light path flow cell and set up between 200 and 300 nm wavelength. Data acquisition and analysis were performed by a PC using the ChromQuest software package provided by the HPLC manufacturer. Assignment and calculation of the compounds of interest in chromatographic runs of biological fluid extracts were carried out at 206 (nitrite and nitrate), 234 (creatinine), or 260 (purines, pyrimidines, ascorbic acid, MDA) nm wavelengths by comparing retention times, absorption spectra, and areas of peaks with those of peaks of chromatographic runs of freshly prepared ultrapure standard mixtures with known concentrations.

### 2.3. Statistical Analysis

All variables were skewed and, therefore, were log-transformed to approach Gaussian distribution before application of parametric tests. Differences between controls and MS patients were assessed by the Student's *t*-test for unpaired observations. Due to the different number of subjects, differences among subgroups of MS patients on EDSS or on clinical MS subtypes (RR, SP, PP) were assessed by the Kruskal-Wallis one-way ANOVA by ranks. A value of *P* < .05 was considered significant.

## 3. Results

The characteristics of both the MS patients and the control group are summarized in Table 1. The clinical classification indicated that 66.5% of the patients were RR, 25.3% were SP, and 8.2% only were PP. 

### 3.1. Serum Metabolic Profile of MS Patients: Purines, Pyrimidines, and Creatinine

**Figure 1:**
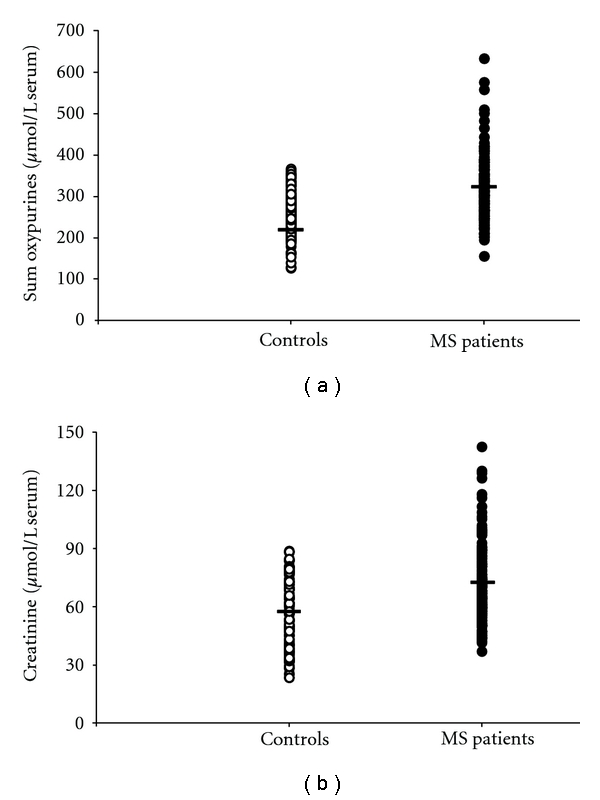
Scatter plot showing the sum of oxypurines (uric acid + hypoxanthine + xanthine) (a) and creatinine (b) recorded in serum of 163 healthy controls and 170 MS patients. Horizontal bars indicate the mean values calculated in the two groups.

**Figure 2:**
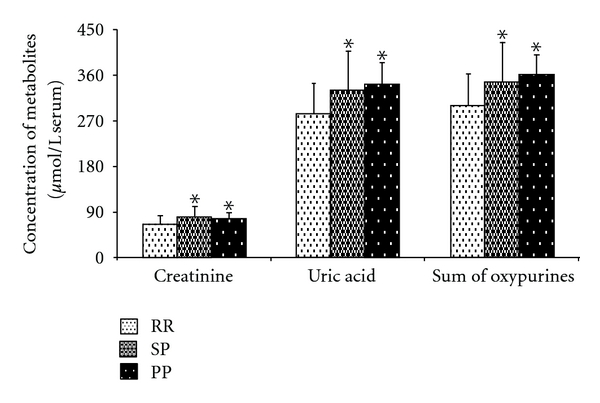
Bar graph showing the mean values of creatinine, uric acid, and sum of oxypurines (uric acid + hypoxanthine + xanthine) in the 170 MS patients divided on the basis of the clinical MS subtype. RR: relapsing remitting; SP: secondary progressive; PP: primary progressive. Standard deviations are indicated by vertical bars. Asterisk = significantly different from RR (*P* < .01).

Data referring to the circulating levels of the different metabolites under evaluation in controls and MS patients are reported in Table 2. With respect to values in controls, the HPLC analysis of serum oxypurines evidenced a 2.94 ± 1.14-fold increase (mean ± standard deviation) in the value of hypoxanthine (*P* < .001), a 2.80 ± 1.53-fold increase (mean ± standard deviation) in the value of xanthine (*P* < .001) and a 1.16 ± 0.27-fold increase (mean ± standard deviation) in the value of uric acid (*P* < .001). When considering the sum of circulating oxypurines in MS patients (316.20 ± 72.21 *μ*mol/L serum; mean ± standard deviation), a 1.21 ± 0.28-fold increase (mean ± standard deviation) with respect to controls (261.16 ± 48.89 *μ*mol/L serum; mean ± standard deviation; *P* < .001) was observed. These values are illustrated in the scatter plot of Figure 1(a); in the same figure, levels of serum creatinine in controls and MS patients are also reported (Figure 1(b)). Similarly to what observed for oxypurines, value of circulating creatinine in MS patients (71.10 ± 19.27 *μ*mol/L serum; mean ± standard deviation) was 1.25 ± 0.34 times higher (mean ± standard deviation) than that recorded in controls (56.87 ± 17.98 *μ*mol/L serum; mean ± standard deviation; *P* < .001). When MS patients were divided on the basis of the disability, no one of the aforementioned metabolites correlated with increasing EDSS. Differently, the classification of the patients into three subgroups on the basis of the MS subtypes (Figure 2) showed that RR patients had significantly different values of creatinine, uric acid, and sum of oxypurines in comparison to both SP (*P* < .001) and PP patients (*P* < .001).

**Figure 3:**
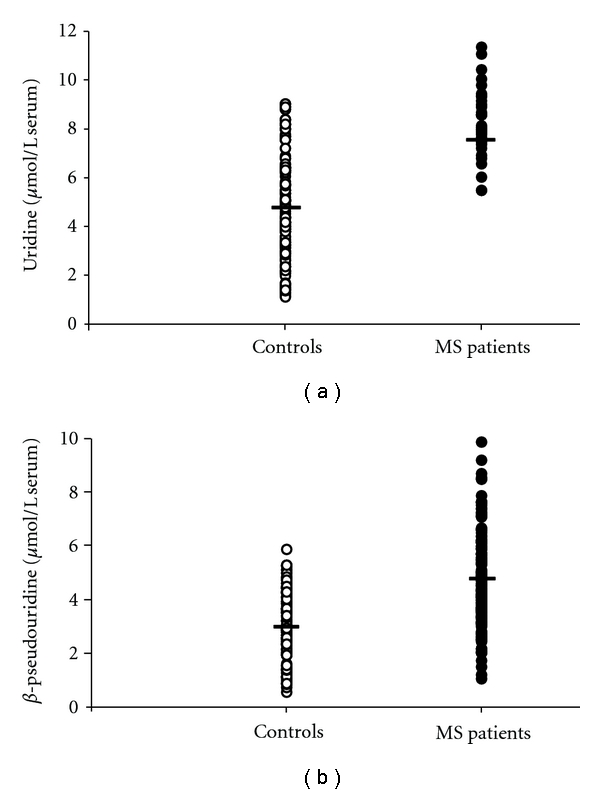
Scatter plot showing the concentrations of uridine (a) and *β*-pseudouridine (b) recorded in serum of 163 controls healthy and 170 MS patients. Horizontal bars indicate the mean values calculated in the two groups.

Among the pyrimidine compounds, uracil, *β*-pseudouridine, and uridine were always detectable in all serum samples analyzed using this HPLC method. Uracil concentration in serum of controls (1.97 ± 0.90 *μ*mol/L serum; mean ± standard deviation) did not differ from that measured in MS patients (2.11 ± 1.04 *μ*mol/L serum; mean ± standard deviation). Viceversa, Figure 3 illustrates that circulating uridine (a) and *β*-pseudouridine (b) were significantly different in MS patients (7.20 ± 1.81 and 4.67 ± 1.71 *μ*mol/L serum, resp.; means ± standard deviations) and controls (4.83 ± 2.19 and 3.03 ± 1.23 *μ*mol/L serum, resp.; means ± standard deviations). Uridine and *β*-pseudouridine did not correlate with increasing EDSS, nor they showed significant differences in the subgroups of patients divided on MS subtypes. 

### 3.2. Serum Metabolic Profile of MS Patients: Oxidants and Antioxidants

**Figure 4:**
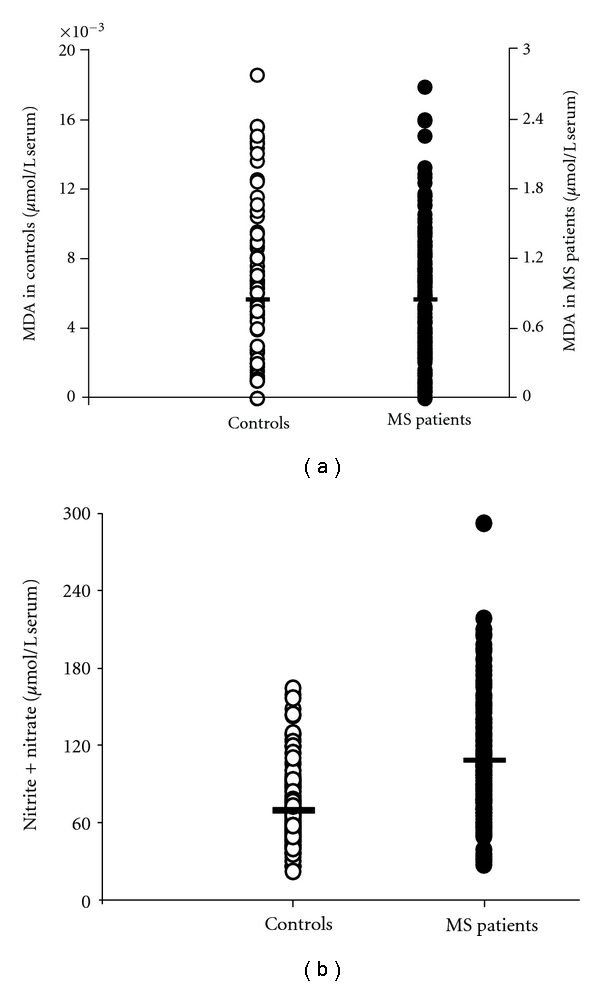
Scatter plot showing the concentrations of MDA (a) and sum of nitrite and nitrate (b) recorded in serum 163 controls healthy and 170 MS patients. Horizontal bars indicate the mean values calculated in the two groups.

Figure 4 reports concentrations of circulating MDA (a), as an index of lipid peroxidation, and of nitrite + nitrate (b), generated from nitric oxide decomposition, in controls and MS patients. MDA in serum of MS patients (0.84 ± 0.53 *μ*mol/L serum; mean ± standard deviation) showed a tremendous 210 ± 132-fold increase (mean ± standard deviation) in comparison with the concentration measured in serum of controls (0.004 ± 0.003 *μ*mol/L serum; mean ± standard deviation). Serum nitrite + nitrate in MS patients (mean ± standard deviation = 107.94 ± 43.87 *μ*mol/L serum) was 1.56 ± 0.63-fold higher (mean ± standard deviation) than the circulating value of these two nitrogen anions measured in controls (69.05 ± 29.04 *μ*mol/L serum; mean ± standard deviation). 

**Figure 5:**
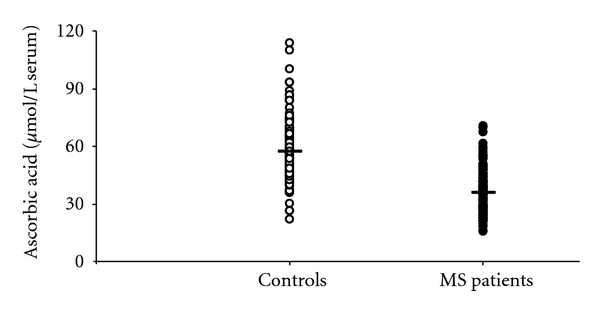
Scatter plot showing the concentration of ascorbic acid recorded in serum 163 healthy controls and 170 MS patients. Horizontal bars indicate the mean values calculated in the two groups.

Data in Figure 5 show that the serum concentration of ascorbic acid in MS patients (37.36 ± 10.95 *μ*mol/L serum; mean ± standard deviation) was 1.54 ± 0.45 times lower (mean ± standard deviation) than that recorded in controls (57.52 ± 14.81 *μ*mol/L serum; mean ± standard deviation), thereby indicating a decrease in this circulating antioxidant as a consequence of the increased oxidative/nitrosative stress occurring in MS. It is worth recalling that MDA, nitrite + nitrate, and ascorbic acid did not correlate with increasing EDSS, nor they showed significant differences in the subgroups of patients divided on MS subtypes.

## 4. Discussion

Data reported in the present study confirm our previous findings obtained in a smaller group of MS patients [16, 30] and indicate alterations of circulating compounds related to energy metabolism, oxidative/nitrosative stresses, and antioxidant status occurring in multiple sclerosis.

Most of the studies suggest that circulating concentrations of uric acid, which metabolically derives from catabolism of phosphorylated purines (ATP and GTP) and also from nucleic acid degradation, are decreased in MS patients [22–25]. However, a meaningful body of the literature contrasts this evidence, indicating that MS patients have levels of plasma/serum uric acid comparable or higher than those recorded in controls [26–29]. In the present cohort of 170 MS patients, confirming our previous observations [16, 30], we again found higher concentrations in serum uric acid than those recorded in 163 age- and gender-matched healthy controls (Table 2). This increase in serum uric acid was accompanied by an almost three times raise in both hypoxanthine and xanthine, thus rendering more evident the overall net increase in circulating oxypurines associated with MS (Figure 1(a)). Furthermore, although none of the aforementioned parameters correlated with EDSS, either uric acid or the sum of oxypurines did correlate with the MS clinical subtypes, with the RR subgroup showing lower values than those found in both the SP and the PP subgroups (Figure 2). 

The three oxypurines considered are mainly produced along the cascade of purine nucleotide degradation, when energy metabolism does not satisfy the cell/tissue ATP demand [34, 35]. Hence, it is conceivable to affirm that MS patients might suffer from imbalance between energy production and consumption. Since the machinery to ensure adequate ATP production is localized in mitochondria, the recent data showing neuronal mitochondrial malfunctioning in MS [12] corroborate the hypothesis that the increase in circulating oxypurines in our patients is the direct consequence of altered mitochondrial functions. Certainly, our results do not support the notion sustained by various studies which affirm that MS patients have lower plasma/serum uric acid than controls [22–25]. In particular, since MS patients suffer from increased oxidative/nitrosative stress [6, 7], it has been suggested that decrease of circulating uric acid in MS is due to the potent uric acid scavenging activity towards peroxynitrite [9]. Together with the results on the full profile of serum purine compounds, our data evidenced a decline in circulating antioxidant defenses of MS patients, in terms of ascorbic acid and not of uric acid decrease (Figure 5). Ascorbic acid, a hydrophilic low-molecular-weight antioxidant, is not synthesized by the human body and adequate amount should, therefore, be assumed with the diet to allow a reasonable distribution by the blood stream to the different tissues. The brain has a specific transporter for ascorbic acid devoted to permit that this compound can cross the blood brain barrier and is accumulated within the cerebral cells, against a concentration gradient [36, 37]. Through this facilitated transport mechanism, cerebral ascorbic acid reaches the concentration of about 2300 nmoL/g wet weight (corresponding to about 2500 *μ*mol/L brain water) and is the second most abundant, water-soluble, brain antioxidant [38, 39]. Ascorbic acid has the same affinity for peroxynitrite than that of uric acid [9], but in the brain it is about 1000 times more concentrated than uric acid [16, 40]. Even if cerebral uric acid had a role as an antioxidant, it appears evident that in the case of increased oxidative/nitrosative stress a decrease in brain ascorbic acid rather than in uric acid would certainly occur. Cerebral uric acid would be oxidized only when the concentration ratio ascorbic acid/uric acid in the brain were in favor of uric acid. According to the present results, our cohort of MS patients, in consequence of increased oxidative/nitrosative stress (Figure 4), showed a 35% decrease in circulating ascorbic acid. Such a decrease might render less efficient the mechanism of its cerebral accumulation and to reduce, in turn, the brain antioxidant capacity. If the decrease in serum ascorbic acid was hypothetically mirrored by an equal decrease in the brain tissue, cerebral ascorbic acid would then be 1400–1500 nmoL/g wet weight, that is, still 700 times higher than brain uric acid [16, 40]. Therefore, it appears that even in conditions of increased oxidative/nitrosative stress, there are not the biochemical presuppositions to sustain a role of uric acid as a valid brain tissue antioxidant, nor to imagine that MS might provoke its decrease in serum.

The evidence of impaired energy metabolism in our MS patients was also supported by data referring to circulating uridine, the value of which was 1.5 times higher than that found in controls (Figure 3(a)). According to previous observations [41], the increase in plasma uridine can be considered as an indirect indicator of tissue energy crisis. In fact, in conditions of metabolic energy imbalance in humans, it has clearly been demonstrated a close association between myocardial ATP exhaustion and the increase either in circulating purines (hypoxanthine, xanthine, uric acid), or in circulating uridine [42]. This reinforces the concept that changes in plasma uridine reflect changes in cell/tissue energy metabolism. The overall conclusion, when analyzing results of circulating purines and pyrimidines, is that MS patients suffer indeed from energy deficit, probably in consequence of altered mitochondrial functions [12, 43, 44]. Since MS patients are at risk of a number of intercurrent systemic inflammatory or noninflammatory conditions [45, 46], it cannot be excluded a significant extracerebral contribution in the overall serum increase of these metabolites. In addition, the muscular involvement in MS [47, 48], possibly caused by a metabolic imbalance of myocytes and also recently evidenced by an increased cost of walking in patients with mild disability [49], might further contribute to exacerbate alterations in the serum metabolic profile of these patients. Data indicating higher serum creatinine in MS patients than in controls (Figure 1(b)) strongly reinforce this concept.

In our MS patients, a significant increase in serum *β*-pseudouridine was also observed. Since this modified pyrimidine is exclusively found in transfer and ribosomal RNAs, its increase in body fluids is generally considered as an index of increased rate of RNAs turnover, due to increased rate of protein synthesis [50]. Since in experimental autoimmune encephalomyelitis (EAE) protein synthesis has been shown to increase 4-fold over the basal level [51], it may be hypothesized that this phenomenon is responsible for the increase in serum *β*-pseudouridine in MS patients. 

In this study, the most dramatic change associated with MS occurred to serum MDA (Figure 4(a)). This compound, originating from the irreversible decomposition of peroxidized polyunsaturated fatty acids of membrane phospholipids, is considered a reliable indicator of increased oxidative stress [52, 53], if properly assayed. In MS patients, the 210-fold increase of MDA over the value recorded in controls is the clear evidence that reactive oxygen species-mediated lipid peroxidation is operative under this pathological condition. Since we also found a significant increase in nitrite + nitrate in serum of MS patients (Figure 4(b)), we can conclude that these patients are exposed to the concomitant oxidative/nitrosative stress, stating that the sum of these two nitrogen anions is considered as an index of NO generation [54, 55]. This implies an elevated risk of producing the highly oxidizing radical peroxynitrite ONOO**^.^** with serious consequences for the brain tissue integrity.

The main limitations of this study are that changes in serum metabolites failed to correlate with EDSS, probably because of a low number of subjects in several patient subgroups. Even the differences recorded for some metabolites when patients were divided into the three clinical MS subtypes failed to discriminate SP from PP, most likely because of the limited number of PP patients. Recruitment of additional MS patients is in progress.

## 5. Conclusions

In conclusion, our results on the serum metabolic profile in MS clearly indicate that these patients suffer from a profound purine and pyrimidine dysmetabolism, potentially due to altered mitochondrial functions. This causes the increase in circulating uric acid, hypoxanthine, xanthine, creatinine, *β*-pseudouridine, and uridine, with creatinine, uric acid, and sum of oxypurines being in correlation with the clinical MS subtypes. The clear evidence of concomitant oxidative/nitrosative stress suggests that possible therapeutic approaches aimed to improve cerebral mitochondrial functions and neuronal energy state, as well as to increase the brain antioxidant defenses, might ameliorate the status of MS patients.

## Figures and Tables

**Table 1:** Clinical features of MS patients and controls.

	Controls	MS patients
Number of patients	163	170
Female : male	106 : 57	115 : 55
Average age at onset	NA	31.77 ± 11.72
Average age at assessment	43.45 ± 3.21	45.27 ± 6.80
Duration of pathology (years)	NA	13.5 ± 5.22
RR	NA	113
SP	NA	43
PP	NA	14

Average EDSS	NA	3.26 ± 2.29

NA: not available.

RR: relapsing-remitting MS; SP: secondary progressive MS; PP: primary progressive MS; EDSS: expanded disability scale score.

**Table 2:** Concentration of circulating creatinine, pyrimidine (*β*-pseudouridine and uridine), oxypurines (hypoxanthine, xanthine and uric acid) malondialdehyde (MDA), nitrite and nitrate (NO_2_ + NO_3_), and ascorbic acid determined by HPLC in serum samples of healthy controls and MS patients.

	Controls (*n* = 163)	MS patients (*n* = 170)
Creatinine	56.87 ± 17.98	71.10 ± 19.27^a^
Uracile	1.97 ± 0.90	2.11 ± 1.04
*β*-pseudouridine	3.03 ± 1.24	4.67 ± 1.71^a^
Uridine	4.83 ± 2.19	7.20 ± 1.82^a^
Hypoxanthine	4.19 ± 1.58	12.30 ± 4.84^a^
Xanthine	1.44 ± 0.96	4.03 ± 2.20^a^
Uric acid	258.08 ± 50.39	299.88 ± 70.17^a^
MDA	0.005 ± 0.004	0.84 ± 0.54^a^
NO_2_ + NO_3_	69.06 ± 29.04	107.94 ± 43.87^a^
Ascorbic acid	57.52 ± 14.81	37.36 ± 10.95^a^

Values are means ± standard deviations and are expressed in *μ*mol/L serum.

^a^significantly different from controls (*P* < .001).
